# Pitfalls in the lab assessment of hypopituitarism

**DOI:** 10.1007/s11154-024-09881-1

**Published:** 2024-04-13

**Authors:** Katharina Schilbach, Martin Bidlingmaier

**Affiliations:** 1 Medizinische Klinik und Poliklinik IV, LMU Klinikum, München, Germany; 2 Deggendorf Institute of Technology, Deggendorf, Germany

**Keywords:** Analytical variability, Interference, Confounders, Specificity, Hormone measurements, Normative data, Decision limits, Biological variables

## Abstract

The diagnostic approach to hypopituitarism involves many disciplines. Clinical symptoms rarely are specific. Imaging techniques are helpful but cannot prove the specific functional defects. Therefore, the definitive diagnosis of pituitary insufficiency is largely based on laboratory tests. However, also laboratory methods come with inherent limitations, and it is essential for the clinician to know and recognize typical pitfalls. Most factors potentially impairing the quality of hormone measurements are introduced in the preanalytical phase, i.e. before the hormones are measured by the laboratory. For example, the timing of blood drawing with respect to circadian rhythm, stress, and medication can have an influence on hormone concentrations. During the actual analysis of the hormones, cross-reactions with molecules present in the sample presenting the same or similar epitopes than the intended analyte may affect immunoassays. Interference can also come from heterophilic or human anti-animal antibodies. Unexpected problems can also be due to popular nutritional supplements which interfere with the measurement procedures. An important example in this respect is the interference from biotin. It became only clinically visible when the use of this vitamin became popular among patients. The extreme serum concentrations reached when patients take it as a supplement can lead to incorrect measurements in immunoassays employing the biotin-streptavidin system. To some extent, hormone analyses using liquid chromatography mass spectrometry (LCMS) can overcome problems, although availability and cost-effectiveness of this method still imposes restrictions. In the post-analytical phase, appropriateness of reference intervals and cut-offs with respect to the specific analytical method used is of outmost importance. Furthermore, for interpretation, additional biological and pharmacological factors like BMI, age and concomitant diseases must be considered to avoid misinterpretation of the measured concentrations. It is important for the clinician and the laboratory to recognize when one or more laboratory values do not match the clinical picture. In an interdisciplinary approach, the search for the underlying cause should be initiated.

## Introduction

The diagnosis or exclusion of pituitary insufficiency has significant consequences for the patient. Unfortunately, clinical symptoms often are not specific. Insufficiencies of hormone axes can exist even without a clear clinical picture, but symptoms can also be wrongly attributed to insufficiencies but have other causes. In addition, an increasing number of patients must be examined not because of clinical suspicion of hypopituitarism, but because the increasing quantity and quality of MRIs performed for other indications (e.g. headache or neurological symptoms) reveals an incidentaloma. Unambiguous identification of hypopituitarism, and the potential need for specific treatment, is essential from a clinical perspective, but also important from an economic point of view.

Biochemical analyses are an important cornerstone to guide the diagnostic process. However, laboratory data also must be used with an appropriate understanding of potential limitations. Causes for misleading laboratory data in the evaluation of hypopituitarism can be found at any stage of the process, from the pre-analytical, the analytical and the post-analytical phase. In the following, we discuss some typical pitfalls related to biological, pharmacological, and analytical factors with the potential to affect the measurement and interpretation of hormone concentrations.

## Preanalytical conditions

Appropriate and standardized preanalytical conditions are key to make laboratory tests a clinically useful tool - way more important than the choice of the one or the other analytical method to measure a hormone within the laboratory. In fact, elegant studies demonstrated that 50–70% of incorrect lab results are caused by factors introduced before the biochemical analysis in the laboratory starts [1]. Therefore, it is important in a clinical setting to take care of critical aspects during the pre-analytical phase. Important considerations for this phase include a wide spectrum of conditions such as the selection of the patient population to be tested, the actual test to be ordered, or the dynamic test to be performed, the preparation of the individual patient, the organization of appropriate sampling conditions, the selection of the sample matrix, and sampling device, and adequate transport of samples to the laboratory. In addition, the laboratory must ensure appropriate preparation and storage of the sample before analysis.

To understand the nature of the potential pitfalls from the pre-analytical phase, a distinction is useful between factors or conditions modifying the concentration of the analyte (in our case: the hormone) within the patient, and factors or conditions leading to “technical” problems in the correct quantification of the analyte in the laboratory. In other words, biological conditions, and variables in vivo and analytical interference in vitro both can cause problems in the interpretation of pituitary hormone concentrations reported by the laboratory. For obvious reasons, some biological variables such as age and gender cannot be standardized. Some, like phase of the menstrual cycle, time of the day, or stress during blood sampling, can partly be influenced, but at least can be documented to allow adjustment of the interpretation of the results during the post-analytical phase. Many in vitro factors potentially interfering with the technical process of hormone quantification, such as additives in the collection tube, sample matrix, the presence of hemolysis, and temperature during transport usually can be standardized, and problems can be avoided in a clinical context. This might require resources, and additional training of staff involved at various departments, but is of great importance. It must be noted that most of these problems caused in vitro cannot be corrected anymore at later stages. At best – if the problem is detected upon arrival at the laboratory – is excluded from analysis because it is impossible to obtain reliable and useful information from analysis. In a worst case - if the error is not detected, but interpretation is affected – it can be harmful for the patient.

The specific details for the best pre-analytical procedures to collect and handle blood samples depend on the specific local setting, and the analytical methods used by the laboratory. Therefore, we abstain from giving general recommendations, and emphasize each laboratory must define such conditions for their clients. However, in the following, we describe some typical examples for biological variables affecting interpretation of hormone concentrations in the diagnosis of pituitary insufficiency. These “pre-analytical conditions” should be reflected by each endocrinologist ordering hormone analyses in the context of diagnosing pituitary insufficiencies.

### Circadian rhythm

Many hormones are affected by a circadian rhythm. The extent of the clinical relevance of the changes in concentrations during the day vary greatly. However, it should be noted that reference intervals for hormone concentrations provided by laboratories often come from studies not necessarily mirroring the timing for blood sampling encountered in clinical practice. Therefore, it is recommended to generally document the time point of taking blood samples for hormone determinations, and to adjust interpretation of the results accordingly. It is well known that particularly Adrenocorticotropic Hormone (ACTH) and cortisol are subject to a pronounced diurnal rhythm. The highest cortisol concentrations are found in the early morning hours and gradually decrease until midday [2]. There may be a slight increase in the afternoon followed by a cortisol decrease to its lowest levels around midnight. The circadian changes of prolactin are similar, however, without specific stimulation there is usually no increase in the afternoon [3]. Growth hormone is secreted in pulses and is greatly affected by the sleep-wake rhythm. During the night, pulsatile secretion shows the highest amplitude, particularly in the first hours of sleep [4]. A change of the day-night rhythm, for example in shift workers, impairs the secretion pattern and leads to lower nocturnal pulse amplitudes [5]. The concentration of thyroid-stimulating hormone (TSH) rises in the early evening, peaks in the early morning hours, and decreases throughout the day [6]. Sleep deprivation causes a greater increase in TSH, which can lead to significantly higher concentrations in the morning [7]. TSH determinations should therefore only be carried out after a normal night’s sleep. The free thyroid hormones (FT3 and FT4) show a less pronounced circadian pattern, with FT3 showing slightly greater fluctuations than FT4 [8]. In men, testosterone, and sex hormone-binding globulin (SHBG) reach peak concentrations in the morning, gradually decrease throughout the day, and rise again from the evening onwards. Clinicians should be aware of this rhythm to avoid misinterpretation of lower testosterone concentrations when samples are taken in the late afternoon. The changes are particularly pronounced in younger men and become less relevant with age [9].

### Stress, acute illness, and body position

Stress is a physiological reaction to physical and/or psychological circumstances. In most cases, stress is also perceived subjectively and accompanied by changes in the vegetative status of the individual. It is almost always accompanied by changes in hormone secretion, and therefore circulating hormone concentrations. Acute illnesses always represent a state of stress and therefore can cause respective changes in pituitary hormones. In contrast, body position or postural changes per se are less important for regulation of pituitary hormones. While these factors significantly affect concentrations of other hormones like renin, or catecholamines, the impact on concentrations of most pituitary hormones is negligible. Nevertheless, a standardized resting phase in a seated position before taking samples for determination of pituitary hormones might be helpful to reduce stress, and thus to standardize sampling conditions.

First and foremost, acute stress is accompanied by an increase in catecholamines, but very quickly followed by changes in secretion of pituitary hormones such as ACTH (and subsequently cortisol), GH, prolactin and TSH [10–14]. Therefore, stress should be avoided during blood sampling to get a realistic impression of baseline hormone secretion. In cases of suspected pituitary insufficiency, very high baseline hormone concentrations induced by stress might give a first hint that insufficiency is unlikely. However, in view of the physiological fluctuations, most guidelines recommend standardized dynamic tests to rule out an insufficiency. Such tests make use of the fact that pituitary hormones respond to stress, and mimic stressful situations in a standardized manner. For example, stimulation tests for the corticotropic axis can be done by injection of the stress hormones CRH and ACTH directly. A less specific, but strong stressor, affecting more hormone axes, is the induction of direct or delayed hypoglycemia in the insulin tolerance or the glucagon test. A normal response in these tests is defined by an appropriate increase in ACTH, cortisol and GH concentrations [15, 16]. It must be noted that the definition of a normal test response commonly refers to an absolute or relative increment in circulating hormone concentrations. Preceding stress, or preceding stimulation tests performed shortly before on the same day, have the potential to significantly affect the baseline value of the respective pituitary hormones, and might affect the secretory capacity of the pituitary for the next stimulus. This might affect interpretation of the observed change in hormone concentration, and applicability of published cut-off concentrations for these tests.

### Medication

A variety of drugs can influence concentrations of pituitary hormones acutely, or chronically. Therefore, a thorough documentation of the patients actual and previous medication is crucial. Most clinicians will be aware that therapies with thyroid hormones, glucocorticoids (i.e. hydrocortisone), testosterone or estradiol all may lead to a suppression of the pituitary hormones of the corresponding axis. However, it is also important to ask patients about intake or application of topical or over-the-counter medication, and, potentially, abuse of illicit substances. Steroids and opioids are particularly relevant when evaluating the gonadotropic and corticotropic axes, as they can lead to insufficiencies [17]. Opioids and cocaine can induce an increase in GH and prolactin. Drug history plays an important role in the assessment of hyperprolactinemia in any case, as various groups of drugs (e.g. neuroleptics, antidepressants, gastroenteric medication, estrogens) can lead to an increase in prolactin concentration [18].

During the investigation of the somatotropic axis, the potential intake of oral estrogens must be considered for the interpretation of GH and IGF-I concentrations. The first past effect on the liver leads to growth hormone resistance and thus to an increase in GH and a reduction in IGF-I concentration [19]. In addition, there is increase in secretion of GH mediated through hypothalamic and pituitary estrogen receptors [19]. Testosterone can cause an increase in GH secretion, mainly indirectly through conversion of testosterone to estradiol by aromatases [19]. The influence of sex hormones on GH secretion is also relevant when evaluating the growth hormone axis in childhood and adolescence. Priming may be needed before testing in order to fully exploit the potential of the growth hormone axis in an individual patient [20].

When assessing the thyrotropic axis, knowledge about the intake of lithium is relevant. Lithium is an effective drug that has been used for decades in the treatment of bipolar disorders. Taking lithium can lead to changes in thyroid function, potentially resulting in both hyper- and hypothyroidism [21]. However, lithium therapy should never be paused without consulting the treating psychiatrist.

It is generally recommended that, if the medication cannot be stopped, it should be documented so that the physician assessing the laboratory can include it in the evaluation of the measurements.

## Analytical pitfalls

As mentioned before, the actual measurement of the hormone concentration - the technical part of the quantification of hormone concentrations in a sample – is only one part in the total diagnostic process associated with term “lab assessment”. The pre-analytical phase as well as the post-analytical phase are at least equally important for the quality of the information we can obtain from a lab test ordered in the context of evaluating suspected pituitary insufficiency.

Overall, significant progress has been made over the past decades in our ability to measure hormones. Chemically, the hormones secreted by the pituitary gland are peptides or proteins. Quantification of such molecules in the clinical laboratory typically is done by immunoassays. In the scientific literature, some assays using other techniques like mass spectrometry (MS) have been described, but they are rarely used in clinical routine. Main reasons are that MS in general is more suitable for smaller molecules, and, compared to immunoassays, measurement of proteins by MS requires more expensive equipment, more complex and time-consuming procedures and sophisticated training for the lab team. Furthermore, currently no studies have proven a significant benefit from using mass spectrometry to measure larger proteins in clinical routine. The situation is slightly different when measurements of peripheral components of the pituitary hormone axes are considered: Particularly for the chemical class of steroids (i.e., gonadal or adrenal hormones), some laboratories have introduced liquid-chromatography mass-spectrometry (LCMS) as a routine technique. For these small molecules, mass spectrometry assays can achieve superior specificity, and have been shown to be less prone to interferences than immunoassays. Nevertheless, even for steroids today immunoassays are by far the most frequently used method in routine laboratories. The introduction of commercially available, automated, high-throughput immunoassays today mostly has replaced local in-house tests, and hormone analyses which had often been done by endocrinologist themselves today mostly are done by large, centralized laboratories. This process was associated with improvements in availability, speed, sensitivity, and reproducibility of lab tests, and in some cases led to better standardization and comparability of results across laboratories. At the same time, the specific interest in hormones became less obvious in the units doing the analyses, and the knowledge about the analytical methods (assays) for hormone determinations, and the awareness for potential pitfalls associated with them, became less present among clinicians. Notably, despite all the progress made in laboratory medicine, all hormone assays have inherent problems [22]. In the endocrine laboratory, such problems typically are related either to factors which are physically present in the patient (in vivo) but interfere with the specific analytical method used in the laboratory, or to difficulties in standardization of the analytical methods themselves, making absolute concentrations, applicability of published diagnostic cut-offs or reference intervals and thus interpretation less reliable. Examples for the first group of potential errors – which originate from the pre-analytical phase but become a problem only in the analytical phase - include cross-reactions, interfering antibodies, and, to an increasing extent, also interfering nutritional supplements, and are discussed in the following. Problems with the standardization of analytical methods mainly have an impact on interpretation of the assay results, and therefore will be discussed in the chapter about the post-analytical phase.

### Cross-reactions

Immunoassays make use of the ability of high-affinity antibodies to specifically bind to distinct three-dimensional structures on the surface of molecules. These structures – the “epitope” of the respective antibody – ideally should be specific for the molecule intended to be measured by the assay. However, many hormones belong to larger families of molecules with very similar structures. In such cases, entire epitopes or parts of them can be present on different molecules. The presence of identical or very similar binding epitopes on different molecules causes “cross reaction” of the respective antibody used in an assay: The antibody can bind to different antigens (hormones), and the assay cannot discriminate if the signal comes from the hormone intended to be measured, or from a cross-reacting molecule. This can lead to falsely low or falsely high concentrations being reported, depending on the design of the assay. Specificity of hormone assays certainly has increased over the years, mainly because today’s immunoassays often use monoclonal rather than polyclonal antibodies, and because many assays employ a sandwich design requiring binding of two different epitopes by specific antibodies before a signal is generated. However, at the same time, to increase throughput in the laboratory, modern commercial assays have skipped complex purification procedures like extraction, which had been used in the past to enhance specificity. Therefore, although modern assays usually come with information on the cross-reactivity of some important substances tested, cross-reactions never can be completely ruled out. This is particularly relevant in situations where extremely high concentrations of potentially cross-reacting substances are present. This can occur in patients with hormone producing tumors or enzyme defects in steroid biosynthesis. Obviously, cross-reaction has also be considered in patients taking drugs which have a structure similar to endogenous hormones. Examples are cross-reaction of the growth hormone receptor antagonist pegvisomant in GH assays [23] or of the aromatase inhibitor exemestane in estradiol assays [24]. Cross-reactions of potential relevance during assessment of insufficiencies of pituitary axes are listed in Table 1. Generally, if results from the laboratory do not fit with the clinical picture at all, cross-reaction might be suspected. In such cases, the measurement should be repeated with another assay method – ideally known to not exhibit the corresponding cross-reaction.

**Table 1:** Examples of cross-reactions in immunoassays relevant for investigation of pituitary function

Analyt	Potentially clinical relevant cross-reacting molecule	Effect
LH	hCG*	LH false positive in pregnancy
GH	Placental GH (GH-V) Pegvisomant (GH receptor antagonist)	Pituitary GH falsely elevated during pregnancy Depending on assay falsely low or high GH concentrations during therapy
Cortisol	Hydrocortisone 21-Desoxycortisol Prednisolon Corticosteron Cortisone Prednisone	Cortisol false positive
Testosterone	Endogenos or exogenous steroids	Testosterone false positive

*hCG human chorionic gonadotropin

### Interference

In addition to classical cross-reactions, immunoassays, but also other analytical methods used in the endocrine laboratory can also be affected by non-specific interference. While the susceptibility of hormone assays to some non-specific conditions known to potentially interfere with measurements, like hemolysis, lipidemia and bilirubinemia, can be tested before methods come to the market, many other interfering substances are less well defined, or patient specific, or become available and therefore relevant only years after the assay had been introduced. For the measurement of pituitary hormones, interference from several endogenous molecules or exogenous substances has been described. If present in a patients’ sample, these substances can affect the hormone detection process, or the process of signal generation in the analytical method.

#### Interference by endogenous antibodies

All antibody interferences are caused by the presence of antibodies in the patient specimen that bind to any component of the assay system. Notably, they rarely interact with the analyte (i.e. the hormone) itself. Most of the interferences from endogenous antibodies actually result from direct interaction with the “tools” used to measure the hormone, namely the antibodies used in the immunoassay, or with components of the mechanism generating the signal in the assay. Such interference can cause false high or false low results, depending on the assay design. Based on their origin and properties, such interfering antibodies are classified as heterophilic antibodies, human anti-animal antibodies or, less important in this context, autoantibodies.

*Heterophilic antibodies* are endogenous, non-specific antibodies that bind to a variety of different antigens – some of them can bind the agent generating the signal in the assay (e.g. anti-ruthenium antibodies or anti-streptavidin antibodies) [25, 26]. They are found, for instance, in patients with autoimmune diseases (i.e. rheumatoid arthritis (RA), where rheumatoid factors may act as heterophilic antibodies) [27]. However, they may also occur in other inflammatory diseases as well as in healthy individuals. Interestingly, interference from heterophilic antibodies is more common in sandwich assays than in competitive immunoassays, and assays with monoclonal antibodies are particularly sensitive to heterophilic antibodies. In particular, ACTH assay have been described to be affected [28]. It can be assumed that the increasing use of antibodies for therapeutic purposes might aggravate the problem in the future [29].

*Human anti-animal antibodies* (HAAAs) are antibodies directed against antigens of other species. The most relevant HAAAs potentially affecting reliability of immunoassays are human anti-mouse antibodies (HAMAs), as most monoclonal antibodies used in these assays are generated in mice. When determining a hormone by immunoassay, HAAAs present in the patient’s sample may bind to the antibodies used in the assay. This can either prevent the antibodies to specifically bind the analyte, leading to a falsely low signal, and concentration. Or, alternatively, the HAAs allow the antibodies in the assay to generate a signal even in the absence of the analyte. The latter typically occurs when HAAAs bind to both, the capture antibody and the detection antibody in a sandwich-immunoassay. The HAAA acts as a linker, resulting in a false positive signal and, consequently, a falsely high measurement result.

If interference from heterophilic antibodies or HAAAs are suspected, “blocking reagents” or “blocking tubes” can be used to pre-treat the samples. However, because concentration and properties of HAAAs can be very different between individuals, the efficacy of such procedures – which are time-consuming and costly – is hard to predict.

#### Interference by biotin

Biotin is a vitamin that is heavily marketed as an over-the-counter dietary supplement and taken by many people due to suggested positive effects on skin, hair and nails, or overall “health”. The doses in many of the dietary supplements marketed today are significantly higher than the daily requirement, and therefore, circulating concentrations in individual patients’ samples today can be very high. Unfortunately, biotin since decades is a crucial component in many commercially available immunoassays [30]. Biotin has a strong affinity for a molecule named streptavidin. Actually, the streptavidin-biotin bond is one of the strongest non-covalent bonds known in biology. This is the reason why the streptavidin-biotin system has been commonly used as part of the signal generation process in immunoassays: Biotin is a small molecule which is used to “label” tracers or antibodies without modifying critical components of their three-dimensional structure. Streptavidin is a large molecule, which easily can be “loaded” with enzymes or fluorescent dyes which are key components of signal generation in enzyme-linked immune-sorbent assays (ELISA), chemiluminescent immunoassays (CLIA) or immune-fluorescence assays (IFMA). In non-competitive sandwich-type assays, high biotin concentrations in a blood specimen can prevent the binding between the biotinylated detection antibody, and the labelled streptavidin. Accordingly, the signal is inappropriately low, resulting in falsely low hormone concentrations being reported. In contrast, in competitive assays, the excess biotin prevents binding of the labelled streptavidin, and thereby detection of the tracer bound to the capture antibody. Because in competitive assays, the hormone concentration in the sample is determined by its ability to displace the tracer, a lower signal corresponds to a higher hormone concentration. Accordingly, in competitive assays, the interference from biotin causes falsely high results for the hormone concentration. This interference is of particular interest for diagnosis of pituitary diseases, because large proteins (like the pituitary hormones) typically are measured by sandwich-type immunoassay, while smaller molecules (like peripheral thyroid hormones, or gonadal and adrenal steroids) typically are measured by competitive assays. Accordingly, the interference from biotin in a sample can mimic typical pathological constellations of the pituitary and the peripheral hormone in an axis that may lead to wrong diagnoses (see Table 2). Most reports in the literature are focus on problems in evaluation of the thyroid axis [31], but other hormone assays can be affected as well [32].

**Table 2:** Miss-leading hormone patterns caused by biotin interference

Pattern caused by biotin interference…	…wrongly classified as
Cortisol ↑, ACTH ↓	Hypercortisolism
E2 bzw. T ↑, LH & FSH ↓	E2- and/or Testosterone-Excess
GH & IGF-I ↓	GH deficiency
fT3 & fT4 ↑, TSH↓	Hyperthyroidism

## Post-analytical phase

The post-analytical phase includes technical aspects (transmission of the measurement results), but also the interpretation of the results. The latter typically is done by comparing an individual patients result to a reference interval or, if dynamic tests are considered, a threshold concentration (“cut-off”). Here, to avoid misinterpretations, two important aspects have to be taken into account: First, the reference intervals or cut-offs must be appropriate for the analytical method used for measurement. Second, particularly in the evaluation of pituitary function, biological variables like age, sex, or concomitant other endocrine or metabolic diseases can significantly modify what can be expected as a “normal” concentration or response. Therefore, the clinical decision limits must appropriately reflect an individual patients’ biological situation. Within this review, it is impossible to comprehensively cover all analytical and biological aspects which define the appropriateness of interpretation of measurement results. However, in the following we provide examples explaining the basic principles.

### Analytical appropriateness of clinical decision limits

Standardization of analytical methods used in the endocrine laboratory is limited. As a consequence, absolute concentrations reported for a single sample by different assays and/or different laboratories, or within the same laboratory on different occasions, can vary greatly [33–35]. One might assume this variation is mainly due to random variations in assay performance, and random errors occurring in the laboratory. However, this is not the case. The main causes for discrepancies between results from different assays are systematic, because they are related to systematic differences with respect to specificity, calibration, or sample preparation. Such differences in absolute concentrations make it inappropriate to uniformly apply the same diagnostic cut-offs or reference intervals to results obtained by different assays [36]. Instead, reference intervals and cut-offs need to be method specific. Unfortunately, such method-specific reference intervals and cut-offs often are not available, and numbers from textbooks or consensus guidelines have to be applied instead. Laboratories and clinicians should be aware of the uncertainty associated with this approach for diagnosis and classification of diseases [37, 38].

### Biological appropriateness of clinical decision limits

#### Background population

The general principle of “reference intervals” is to compare an individual patients’ laboratory results to the results obtained from a background population. However, the appropriateness of the specific background population selected by the assay manufacturer, or the laboratory, for interpretation of the measurement results of an individual patient can be difficult to asses. Sometimes, very limited information is available with respect to the inclusion and exclusion criteria applied. Genetic and ethnic background, geographical region, socio-economic status, nutritional habits, and many other factors can have an influence on the distribution of hormone concentrations in a population. Therefore, the definition of what can be considered normal might vary, depending on the population studied. Examples have been described also for measurements relevant to the diagnosis of pituitary diseases [39–41].

#### Age and sex

It is well known that age and gender must be considered when evaluating measured concentrations for gonadotropins, estradiol and testosterone. Most laboratories automatically provide reference intervals adjusted for such factors. However, not always are method-specific data available to support a good definition of normality for all potentially relevant subgroups. I.e., in women, the phases of the menstrual cycle or the postmenopausal status and, if applicable, an existing pregnancy must also be considered. The latter also applies to prolactin, which physiologically increases during pregnancy with some studies reporting concentrations up to 177 ng/mL (3800 µIU/mL) in their assays [42, 43]. Changes in sex hormones during puberty, menstrual cycle, pregnancy, menopause, and andropause, can also affect concentrations of other diagnostically relevant, pituitary function-dependent hormones. For examples, the above-mentioned changes of the gonadotropic axis are modulating age- and sex-dependent changes in the somatotropic axis [44], and in part explain the characteristic patter of IGF-I concentrations from birth to senescence [44]. Particularly during puberty, gender plays a significant role, with higher levels seen in boys than girls, while the effect of sex on IGF-I concentrations is negligible later in life. With aging, IGF-I loses its diagnostic value with regard to an insufficiency of the somatotropic axis [45, 46]. In childhood, adolescence and young adulthood, the sensitivity of IGF-I is relatively high, whereas it decreases significantly from the mid-30s. The main reason is that low IGF-I concentrations are common even if pituitary function is normal. Therefore, a GH stimulation test is often necessary to reliably detect a corresponding deficit and to establish an indication for GH therapy [47]. Whereas age and sex play a limited role in diagnosing insufficiencies of the corticotropic axis in adults, there are data to suggest that it may well be relevant during childhood [48]. This is also true, and to a greater extent, for TSH, fT3 and fT4, for which different reference intervals apply in childhood, adolescence and puberty, whereas the changes in adulthood are modest [49].

#### BMI

It has long been known that the body mass index (BMI) impacts GH secretion, with higher BMI being associated with lower GH concentrations [50]. The pathophysiological mechanisms are not completely understood, and some obvious factors such as Matsuda insulin sensitivity index (ISI), body fat content and blood lipids are not directly associated [51]. However, in recent years, it became generally accepted that for some dynamic tests of the somatotropic axis, namely for the GHRH-arginine test and the glucagon test, BMI-specific cut-offs must be applied [52, 53]. In contrast, BMI does not appear to have a clinically relevant influence on peak GH concentrations in the insulin-tolerance-test and the macimorelin test [54].

#### Impact of concomitant endocrinological, metabolic and other diseases

In the diagnosis of pituitary insufficiency, it is important to understand that the dysfunction of one of the hypothalamic-pituitary-target organ axes often is associated with dysfunction of the other axes. For example, hypo- or hyperthyroidism can lead to reduced GH secretion [55]. Conversely, GH deficiency alters the conversion of FT3 to FT4, and GH substitution can unmask gonadotropic or thyrotropic insufficiency [56, 57]. Another common example is hyperprolactinemia, which inhibits the hypothalamic gonadotropin-releasing hormone (GnRH) pulse generator, subsequently leading to hypogonadotropic hypogonadism [58]. In females with PCOS, changes in GH, luteinizing hormone (LH) / follicle stimulating hormone (FSH) ratio, estrogens and androgens are to be expected [59]. These kinds of interactions should always be considered during the evaluation of baseline or stimulated hormone concentrations.

As mentioned above, BMI, and metabolic diseases can have an impact on various pituitary hormones or the peripheral components of the respective exes. In men with obesity, increased aromatase activity in adipose tissue leads to increased conversion of testosterone to estradiol. This imbalance between androgens and estrogens in favor of the latter in turn may lead to hypogonadism and increase of body fat content [60].

Several other diseases have potential influence on the hormones of the hypothalamic-pituitary-target organ axis (i.e. renal and hepatic dysfunction). The extent of the impact usually depends on the severity of the disease. When in doubt, the influence of existing comorbidities should always be considered in the case of unclear laboratory findings.

## Data Availability

No datasets were generated or analysed during the current study.
